# The first hematopoietic stem cell transplantation centre in Iraqi Kurdistan: nursing perspectives and education

**DOI:** 10.3332/ecancer.2019.899

**Published:** 2019-01-24

**Authors:** Marta Canesi, Chiara Broggi, Kizhan Fazil, Mardin Jafr, Laura Russo, Valentina Panzetti, Gloria Ciabatti, Giulia De Riso, Andrea Mastria, Hiwa Sadiq Sidiq

**Affiliations:** 1 Fondazione MBBM ASST, Monza, Italy; 2 Hiwa Cancer Hospital, BMT Centre, Sulaymania, Kurdistan; 3 Ospedale V Fazzi, Lecce, Italy

**Keywords:** cancer care, capacity building, clinical supervision, international collaboration/cooperation, multi-cultural issues, nursing, nursing education, nursing leadership, transplantation

## Abstract

**Aim:**

To describe the nursing capacity-building process within the foundation of a hematopoietic stem cell transplantation (HSCT) centre at the Hiwa Cancer Hospital (HCH), Sulaymaniyah, Iraqi Kurdistan, referring to nursing education, empowerment and leadership.

**Methods:**

1) Capacity building, the process by which individuals, organisations, institutions and societies develop abilities to perform functions, solve problems and set and achieve objectives, was implemented; 2) Nurse intervention was based exclusively on training and coaching on site, which is an innovative approach, since more often experts are brought to the centre to train people on site; 3) Nurses’ personal skills, knowledge and training needs in the field at the HSCT centre were preliminarily explored through an online survey, and intervention was also addressed considering personal preferences and challenges; 4) Clinical documentation implementation and nursing professional organisation improvements were developed.

**Results/findings:**

1) up to June 2018, 98 patients have been transplanted (69 autologous + 29 allogeneic graft). The centre at the HCH represents the first in Kurdistan and the only centre carrying out allogeneic transplants in the whole of Iraq; 2) twenty-two staff nurses; three nurses in charge and one head nurse are employed in the HSCT centre. Nurses currently have good capability to manage daily care for patients in the HSCT centre. There are still training needs to be addressed; 3) and 4) implementation of organigram, job description and nursing plans. The situation, background, assessment, recommendation method for nursing handover was introduced. Nursing shifts duration was changed.

**Conclusion/implications for nursing:**

Capacity building cooperation is a powerful means to successfully establish a high technology medical programme, and is a feasible method to enhance skills and expertise even in low resources contexts. The programme is still in progress and consolidating actions are still required. Nurses need to enforce professional leadership and work organisation. The HSCT centre local team needs to improve teamwork and shared decision making.

## Background

Hematopoietic stem cell transplantation (HSCT) is an advanced procedure, performed on patients affected by a variety of haematological and non-haematological diseases, including acute leukaemia, malignant lymphomas, multiple myeloma (MM), thalassemia major (TM) and neuroblastoma. In an autologous transplant, stem cells are collected from the patient himself, frozen, stored and then given back to the patient after a conditioning regimen. In an allogeneic transplant, the patient receives stem cells from another individual who is a genetically matched donor [1]. HSCT and HSCT nursing are evidence-based practises and require a supportive environment that includes professionals and institutional motivation, human resources, technology and services (i.e., radiology, transfusional medicine, labs and infection control). HSCT is also an expensive procedure and many countries (especially in some geographical areas) cannot afford it, due to a lack of resources and expertise. Limited resources mean scarce opportunities for expensive and high technology care [2]. These countries are forced to send patients abroad to undergo HSCT; this creates a psychological and financial burden for families and high costs to the country of origin. Moreover, some of these situations suggest a lack of follow up for patients resulting in less favourable clinical outcomes. It has been proved that HSCT performed in the country of origin is cost saving for both the government and patients [3]. Local access guarantees a better quality of life to patients and families, reducing stressful events such as logistics (the search for a house, transportation), cultural burden (language, cultural and religious different contexts), financial burden and prior psychological distress (loneliness, isolation).

Iraqi Kurdistan (officially named the Kurdistan Region of Iraq) is a region with considerable oil and mineral resources but currently in a deep financial crisis due to the conflict with the Islamic State and the fall of the price of the oil. Iraqi Kurdistan is experiencing a huge immigration with a very high number of Syrian and Iraqi refugees living in the territory. The Italian Ministry of Foreign Affairs (IMFA) has been supporting the Kurdish people with Health and Social Projects. Due to the absence of HSCT activity in Iraqi Kurdistan and the cost to the government of sending patients (especially TM) to India and Jordan for HSCT, in 2015, thanks to the financial support of the Italian Agency for Development Cooperation, an Italian non-governmental organization (NGO) submitted a capacity building proposal for the foundation of the first HSCT Centre in the Iraqi Kurdistan Region. Furthermore, the growing number of refugees has led to an increase in the number of people (especially children) affected by TM, raising the need for transplant activity within the Kurdish borders. In 2016, the project received other funds managed by a different Italian NGO, thanks to a second call of the IMFA—General Direction for Development Cooperation.

Twinning between a centre in a high-income country and one in a low–middle income country (LMIC) can improve patient care significantly. Progress is made despite the critical challenges [2, 4]. One of the main successful concepts in twinning projects is that strategies and solutions must come from the local team thanks to a two-way transfer of expertise, skills and knowledge. Long-term commitment and mutual respect and responsibilities are crucial [5]. Nursing staff are essential in a high specialty field like HSCT where patient care is so crucial: nurses are at the bedside 24 hours a day and have a critical role in monitoring the patient, preventing and managing complications, educating the family and helping them to understand the disease and the plan of treatment. However, in LMICs, there are a number of challenges nurses have to face: inadequate staffing levels, lack of support, limited access to education and unsafe practise environments [2, 6]. Even though the literature about nursing in the blood and marrow transplantation (BMT) field in LMICs is scarce due to the lack of experiences, this issue has been studied in the paediatric onco-haematology area: it is demonstrated that specialised nursing education, adequate equipment and resources are needed to support nurses in complex practises [2]. This also promotes effective communication and partnerships with medical doctors, enhancing the role of nurses as strong healthcare team members and leaders of their own plan of care [2].

This paper is about the foundation of the first HSCT unit in the Iraqi Kurdistan Region, from a nursing perspective and considering the nursing training programme, challenges and future developments.

## Methods

At the end of 2015, an exploratory mission by an Italian medical doctor, an expert in HSCT, was performed at the Hiwa Cancer Hospital (HCH) (located in Sulaymaniyah, Iraqi Kurdistan) in order to evaluate the presence of the essential criteria to start an HSCT activity, as elements and services are always needed in that clinical field (e.g., areas evaluated: personnel, central venous catheters (CVCs), apheresis service, blood bank and immunohematology) [7]. The site was identified as a suitable site to start the programme. This is the leading centre in oncological care in the entire Kurdistan region and one of the first in the whole of Iraq. The hospital operates inpatient wards and outpatient clinics for both children and adults with hemato-oncological conditions. The exploratory mission confirmed the feasibility of the programme based on the essential criteria for transplant activity. A recruitment campaign was delivered and around 40 Italian HSCT experts were enrolled as volunteers (medical doctors, nurses, biologists, lab technicians and physicists). Nurse selection was based on: 1) previous experience in the field; 2) English speaking/comprehension; 3) previous cooperative experience and 4) flexibility, adaptability.

Funding: the project was funded by the Italian Agency for Cooperation Development. Funds were administered consecutively by two Italian NGOs. HCH contributed to the final budget, providing existing instrumentation, laboratory facilities and accommodation for the volunteers.

The capacity building process was chosen as the leading methodology to set up the programme: after experts’ involvement, local professionals were trained at HCH and continuous education was promoted as a strategy to keep the quality of work and care high [7, 8]. This method aims for sustainability and accountability [9].

From the very beginning, efforts were dedicated to staff training: this takes the form of lectures in classrooms, bedside consultations, rounds in the clinics and coaching for focussed on the job training and task shifting [3].

In April 2016, a 3-week educational course was provided to the all HSCT staff. It was delivered in English for the local professionals with an English proficiency between A2 and C1 [10].

Five months after the training, to understand nursing adherence, satisfaction with the educational programme and perceived training needs, staff who were educated completed an anonymous questionnaire online, based on 25 questions (10 multiple choices, 8 open questions, 7 sentences to be commented through a Likert scale 1–5), to evaluate the HSCT programme learning and implementation process. The questionnaire was especially created for this aim, considering the topics of the training course. In addition, the qualitative data were collected through open questions about personal satisfaction and perceived unmet educational needs.

In addition, examination of bedside daily activity in the unit allowed Italian nurses to understand the emerging and existing problems. Interventions were carried out to enhance nursing organisation and leadership.

## Results

### Transplantation activity

Currently, the HSCT unit has eight beds (four double rooms) for pre-HSCT patients (conditioning regimen) and six single rooms for transplanted patients (high-efficiency particulate air filtered). They are all positive pressure rooms.

In June 2016, the first autologous transplant was performed in an MM adult patient (peripheral blood stem cells source). In October 2016, the first allogeneic HSCT was carried out on a child affected by TM. The donor was a compatible sibling (human leukocyte antigen-identical). In May 2017, the first allogeneic HSCT on adults was performed in a patient with acute myeloid leukaemia. Up to June 2018, 98 HSCTs have been performed. Data are illustrated in Table 1. Considering the autologous HSCT: granulocyte engraftment occurred on day + 11 (narrow range from + 9 to + 12); platelets engraftment occurred on day + 12 (range from + 10 to + 17). Among the allogeneic transplanted patients, granulocyte engraftment occurred on (median) day + 17. The main complications developed are a graft versus host disease (GVHD), intestinal perforation (*n* = 1; successful surgical treatment performed), cytomegalovirus (CMV) enterocolitis (*n* = 1), low-grade microangiopathy and posterior reversible encephalopathy syndrome.

All the patients had a tunnelled CVC (Hickman or Broviac line) inserted. Considering adults, all the CVC were removed within 2 months from the HSCT because of the high risk of infection, once at home. In fact, frequently the living arrangements of patients coming from rural areas or even the domestic settings of the ones living in urban areas did not meet the necessary hygienic criteria and the patients or caregivers were not able to take care of the devices or to identify signs and symptoms of infections to be promptly referred to the healthcare team.

### Nursing training

**Figure 1.**
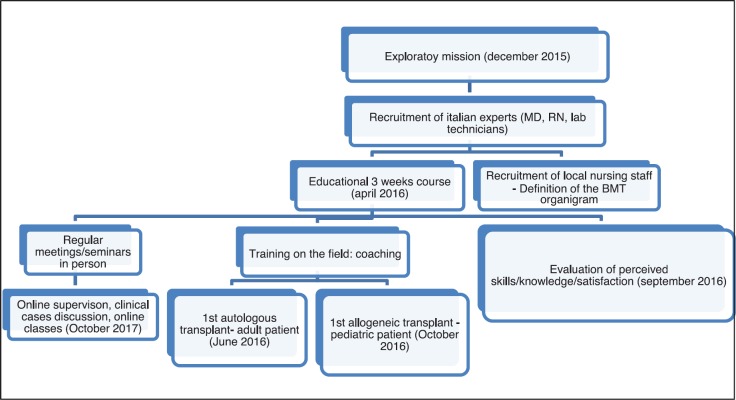
Flowchart of the nursing programme within the start-up of the HSCT centre.

Figure 1 summarises the process involving nurses and their training within the foundation of the HSCT programme at HCH.

The educational programme was developed to provide haematology and HSCT education to the local staff (nurses, doctors and lab personnel). Education sessions were delivered by Italian or local experts in the field. At the end of the course, participants received a certificate of attendance. Content covered in the lectures related to HSCT indications, principles and practise; complications and follow up; infections and stem cells collection and processing [7]. Several dedicated nursing topics were provided: essential haematology and oncology (leukaemia, lymphomas, thalassemia, aplastic anaemia, MM and neuroblastoma); chemotherapy (safe preparation and administration; extravasation); HSCT complications management (i.e., mucositis, GVHD); monitoring vitals and alarm signs; critically ill patient (shock, sepsis); nutritional aspects; infection control (policies and principles); vascular devices management peripherally inserted central catheters (PICC) and CVC line; blood components (standard for administration) and patients/parents education.

In the meantime, verification of clinical protocols dedicated to the transplantation programme was conducted. A preliminary list of nursing protocols has been developed:

- Oral care[11]
- CVC management–no-touch technique[12]
- Food and diet around HSCT
- Chemotherapy extravasation: prevention, management and treatment
- Pain management[13, 14]
- Isolation rules[15]
- GVHD management[16, 17]

Training in the field used a coaching method: for almost the whole duration of the project an expert Italian nurse was in the unit, supervising nurses and addressing their training needs, to enhance knowledge and technical skills. Considerable effort has been made to enhance the teamwork within the HSCT team (apheresis and clinical team; nursing and medical staff). This has been done through regular meetings: morning briefings (every working day), weekly seminars on clinical and scientific issues and transplantation meetings (once a week) with discussion of all the transplantation cases. The morning meetings were regularly attended by the head nurse and at least two of the three nurses working during the previous night shift. About 60% of the nurses attended the other seminars or meetings. The hospital nurse coordinator joined the BMT nursing staff to discuss specific topics requiring his involvement and responsibilities (e.g., venous access and isolation rules). The aim of these meetings was to enforce a decision sharing method and develop a good communication process. Moreover, these meetings enhanced the teamwork strategies because all the clinical professionals (medical doctors (MDs), registered nurses (RNs), pharmacists, lab technicians and apheresis team) attended the morning rounds and the afternoon outpatient clinic have been attended simultaneously by nurses and doctors in charge of the patients. Within the team meetings and the clinical training, a problem-solving attitude was encouraged and, at least partially, developed.

### Nursing considerations and issues in transplant settings

#### Online survey

The online survey dedicated to nurses had an overall response rate of 85% (*n* = 17/20 nurses). Nurses were mainly female (70.6%) with a median age of 23 years. Fifty-three percent of the sample were College Nurses (4-years University Education—bachelor of science in nursing (BSN)) and the others were Diploma Nurses (2-years Education). Almost all of them (94.1%) had previous work experience, mainly in a hospital setting (Emergency Department and Operating Room). Some of them were employed by HCH itself on the oncological units. One of the nurses worked as a pharmacist assistant. Eighty-eight percent of the HSCT staff nurses spontaneously applied to be in the team, motivated by the originality of this field in Iraqi Kurdistan and the opportunity to *‘give patients a second life’.* All the respondent nurses would apply for the HSCT position again. Nurses reported their appreciation of their role in HSCT because of new and challenging technical skills and knowledge (central venous line management, hematopoietic stem cells infusion and interpretation of vital signs), teamwork and nursing responsibilities. On the other hand, they reported *‘responsibilities’, ‘lack of knowledge’* and *‘management of critically ill patients’* as the biggest challenges.

Nurses were asked to rate their perceived knowledge about different topics, illustrated in Table 2. Rates were based on a Likert scale (1–5, best score-highest score). Nurses reported further educational needs as follows:

1. drugs management (82%)
2. critical care in BMT (70%)
3. GVHD and its nursing care (41%)
4. pediatric nursing (30%)
5. devices, particularly referring to non-invasive ventilation techniques and peripherally inserted central catheters (52%)

The results of the *online survey* about training and skills/knowledge showed that nurses perceived a high level of improvements on topics they were already familiar with (i.e., CVC management and vital signs interpretation) while they still needed training about HSCT specificities (i.e., GVHD management, HSCT principles and HSCT complications). Interestingly, 53% of the participants did not feel safe and confident about working with children, because of a lack of skills and experience.

### Nursing documentation

A clinical chart has been implemented with the local nurses and doctors, for daily patient assessment. Charts have been created for data registration: vital signs and fluid balance, drug prescription and administration, pain assessment and other daily events (i.e., fever in neutropenia, blood cultures). Dedicated procedures and documentation have been produced to create an infection control programme.

Nursing handovers tended to be very poor and resulted in fragmented nursing care. Nurses reported struggling with complex patients and the amount of detailed information to be collected and handed to colleagues. A new handover method, Situation, Background, Assessment, Recommendation (SBAR), was introduced and tested with a simulation of clinical cases, nursing handover and filling in the SBAR documentation provided [18, 19]. It still needs implementation, as not all nurses are yet compliant, especially in the absence of Italian supervisors.

### Nursing organisation and leadership

Kurdish health authorities are generally concerned about nursing training, absence of defined nursing competencies, roles and responsibilities. This results in lack of care and inefficient use of nurses in clinical care [20]. Job description and nursing care plans were identified as possible solutions to these gaps: moreover, they were expected to be already developed by 2010 in all of Kurdistan (not only the Iraqi region) but there is still uncertainty regarding their status [20]. Thus, Italian nurses together with the local Head Nurse wrote operational documents such as the Nursing Job Description and Nursing Care Plans.

An organigram, representing the responsibility tree, was depicted and built on a functional organisational concept [21] so that the unique scope and authority of each specialist were made clear and the areas of interest were defined. This led to a substantial modification of the organisation and also to cope with existing international standards. Within the Nursing functional department, three main areas of intervention were identified, each with a responsible nurse coordinating the work plan as follows.

- Outpatient clinic + infection control.
- Inpatients: HSCT unit + nurse educator.
- Inpatients: pre-HSCT unit + new admissions.

Up to October 2018, HSCT nursing staff includes 22 nurses (11 College Nurses + 11 Diploma Nurses), three Lead Nurses and one Head Nurse. International staff considered it to be essential that the nursing staff developed theoretical knowledge in the field and the ability to explain the rationale of its activity.

The hospital direction and the Head Nurses shared this policy and different evaluations were organised up to October 2018. Nurses were evaluated with multiple choice questionnaires to test their knowledge and about nursing and the specific clinical field. Some nurses failed the test and were replaced together with others who were not interested in being involved in the professional activity requiring personal effort, study time and extra shifts.

At the beginning of the transplant activity, nurses were working on a 12/24-hours schedule. Few patients were admitted in the unit and long shifts seemed to be functional, responding to the local personnel’s needs. As time goes by, the clinical activity in the unit has improved, requiring more energy and attention from nurses. Tiredness and a high risk of errors were reported. Thus, in May 2017, nurse shifts were changed to an 8-hour rotation. The introduction of the new rotation caused the resignation of 8 nurses out of 20, who did not want to share the new working times.

### Nursing training perspectives

A technical programme is ongoing to allow nurses to improve their technical skills. It includes a first aid course (delivered by a local medical expert) and a basic life support course (including the obstructed airways management) held by an Italian nurse. High skills to be developed are: PICC insertion and the use of continuous positive airway pressure and noninvasive ventilation [22]

## Discussion

Transplant activity is slowly increasing: local doctors from HCH and other institutions are starting to identify HCH as a referral centre for their own patients. Moreover, a new regulation should be shortly approved so that autologous transplants are no longer authorised nor financially supported by the government if done outside the whole Kurdistan region.

Considering the complexity of this medical field and the related nursing care, these results seem reliable and understandable.

As of June 2018, nurses can autonomously manage routine activity and stable patients in HSCT, especially adults. They still need supervision and advice about unexpected events and how to manage clinical emergencies.

Currently, over 100 documents have been published as official documents of the HSCT unit and included in the quality manual. The list includes procedures, clinical protocols, attachments and forms. Documents belong to functional units within the HSCT unit: collection and processing facilities, blood bank, clinical unit and nursing. Several flowcharts and algorithms (Alg.) have been produced and stuck on the unit walls to be rapidly consulted by staff (i.e., vital signs tool; GVHD evaluation tool; fever Alg.; basic life support and defibrillation Alg. and seizure Alg.).

Some *cultural differences* emerged during the coaching training and the writing of the job description. Kurdish nurses are not used to caring for patients’ personal hygiene and patients do not expect them to do that. A signed agreement, involving the hospital’s general director, was needed to state when the nurses should deliver hygiene and personal care (as in the case for skin inspection for GVHD manifestations) [23]. Another barrier was identified in the isolation rules. Thus, hospitalised Kurdish people are used to being directly cared for by a family caregiver, co-living in the patient’s room 24/7. The caregiver can usually stay in the same room as the admitted patient, without any restriction, even if the room is not for single use. Trainers had to explain the reasons why isolation is needed for transplanted patients. A strategy was identified by allowing family members to visit their relatives through a window, using a balcony, previously closed to visitors. These rules were not applied to children, where a caregiver is mandatory in the room 24/7.

### Nursing policy implications

Nurses are currently the most committed and well-defined professional team in all of the HSCT units and have started to be proactive, suggesting interventions and activities. Some conflict with medical staff is emerging together with the growing abilities and capacities of nursing staff. Nurses would like to be more actively involved in patients’ care plans and they are starting to be independent in making some clinical decisions (e.g., neutropenic fever management, skin care, mucositis treatment, patient and caregiver education). This is new to the local dynamics in the healthcare team and appears to cause discomfort to the medical staff. Moreover, the implementation of a written clinical documentation made nurses more aware of their professional responsibility related to the record of patient conditions and care delivered. This forced doctors to be more careful and precise about written drug prescriptions that were previously incomplete or even absent.

The nursing job description defines criteria to be selected as an HSCT nurse and a training course has been defined. This made BMT nursing, a special field of practise, requiring dedicated competences and skills. This is something unique in the entire Kurdistan region, where specialisation courses exist (clinical master’s degree) but do not have a real influence or application in practise. In the past 14 months, the nursing HSCT staff has changed several times due to all the reasons mentioned above. The training course was, then, entirely run three times by local staff.

Currently, HSCT nurses are recognised as well trained by the entire hospital so they are often consulted for clinical issues. In June 2017, they started delivering courses for nurses and patients/caregivers regarding issues related to chemotherapy. Initially, these were dedicated to the units where neutropenic patients are admitted. Going forwards, the programme will be extended to the entire hospital to share evidence-based protocols and practise.

Nurses reported that working alongside the international staff contributed to making nursing a challenging and gratifying discipline because they had the chance to identify their own competences and responsibilities. They asked for the training about scientific database consultation, critical appraisal of articles and literature review, showing an increasing empowerment and involvement even in higher fields, such as research. The co-operative project supported the hospital offering access to scientific databases and some journal subscriptions. However, information technology still represents a barrier to be faced: Internet connexion is not always available and IT technologies and resources are few. However, good IT consultants work in the hospital and have been in contact with the nursing staff, for the first time.

Less than one-third of the nurses reported limited compliance with 8-hour *shifts*. At the time of writing, the changed implementation of shifts is still very new and probably not yet fully accepted. Some reasons can be identified: first, due to the financial crisis, nurses are not receiving their salary on a regular basis. Many of them need to have a second job to earn a decent salary. The new rotation seems to make this more difficult. Second, transportation challenges were reported: nurses mainly use taxis to reach their workplace. Shorter shifts mean more frequent shifts and higher costs. To solve this problem, the hospital administration has provided the nursing team with a car to be shared, thus taking care of transportation costs. This strategy has been approved by the nursing staff: they are happy about the solution, especially female nurses who were the ones also experiencing a cultural barrier that considers it to be strange and inappropriate for a woman to be out and about by herself during the evening or even the night.

In June 2018, most of the nurses were doing two jobs because of money or professional local policies: due to this, they have to complete a 2 years post-BSN rotation, with paid shifts in different hospitals. They reported being very tired because of these activities and workload. The administration of HCH is trying to reduce the number of rotation hours due and to reward them with a small increase in salary. The situation is still in progress and it is unique: it demonstrates that BMT nursing care is considered to be highly specialised: the idea of a nurse as a professional, with a defined field of competence, is slowly developing among the nurses themselves and the hospital administration.

Reagrding the literature, there are many cooperative and twinning projects involving hemato-oncology in LMICs, in many geographic areas (e.g., Latin America, Africa and Asia). The main highlights of the programme presented in this paper are that the programme permitted the implementation a very high specialised field of care such as BMT, not focussing on primary care pathways that are very much common in the LMICs. In addition, this project was based on training in the field meaning that experts worked side by side with the local team. In this daily shared work, it was possible to promptly identify and manage issues, resources, areas needing implementation, cultural issues and differences, influencing the clinical job (e.g., gender and religious influence on personal care). This is not very common: many cooperative programmes are based on very brief periods of time spent in the foreign country, and mainly the support is provided by online supervision, shared documentation and clinical cases discussion. These strategies are currently used by the team in Kurdistan as well as a final step, before leaving them completely autonomous and just to be available in case of need.

## Conclusion

International cooperation has been successful in setting up the first HSCT centre in Iraqi Kurdistan. The capacity building programme proved to be a feasible method to enhance skills and expertise even in low resource contexts. Nonetheless, the programme is still in progress and improvements and consolidating actions are required.

Nurses still need support and encouragement. They are currently developing a stronger leadership and the capacity to take care of their own professional and educational needs.

Since 2016, HCH has been included in the European society for BMT (EBMT) network giving its staff the chance to be in contact with global experts and greater opportunities.

Between 2016 and 2018, three nurses, three doctors and a biologist won scholarships (sponsored by the EBMT Nurses Group, American Society of Haematology and an Italian NGO, respectively) and received focussed training in different Italian HSCT Centres. Moreover, they had the chance to attend international HSCT meeting and network in their specific clinical field.

HSCT is a highly specialised and challenging field of practise for nurses. This experience demonstrates that high specialty cooperative projects can be promoted and successfully performed in LMICs. Nurses can develop skills and knowledge and be leaders in their own institution, changing the current professional nursing role in their country, being considered as professionals and experts by their teams.

Within this programme, enhancing the expertise of nurses working in BMT also influences nurses working in the oncology, haematology and paediatric unit. In fact, the nursing staff at the HCH is strongly committed to the care of patients and willing to improve the quality of care. Thus, it was possible to change daily practises in the out-patient and in-patients units (e.g., patient care, alarm signs and symptoms, infection control, isolation rules and vascular access). In fact, nursing care in BMT includes many principles of nursing in onco-haematology and patients undergoing HSCT are usually under the supervision of the onco-haematology staff, before they are admitted to a BMT unit. Thus, it was particularly important to try to also align their practise to the latest evidence and the newly created protocols and documentation of the BMT in the same institution.

## Conflicts of interest

The authors declare the absence of any conflict of interest.

## Funding statement

This project was funded by the Italian Development Cooperation Agency (AICS), thanks to the support of two Italian non-governative organizations. It has also been funded by the local institution Hiwa Cancer Hospital.

## Figures and Tables

**Table 1.** HSCT performed ad HCH (June 2016–June 2018).

	Age groups	
	Adults	Paediatrics
Autologous	59	10
Allogeneic	8	21

**Table 2.** Perceived skills and knowledge obtained through an online survey (Likert Scale).

Topic	Score (Likert Scale)				
	1	2	3	4	5
CVC management	0%	11.8%	35.3%	52.9%	0%
Vitals and their interpretation	0%	0%	5.9%	70.6%	23.5%
HSCT principles and indications	11.8%	41.2%	23.5%	23.5%	0%
GVHD and HSCT complications	0%	5.9%	52.9%	41.2%	0%
